# A comparative mapping of plant species diversity using ensemble learning algorithms combined with high accuracy surface modeling

**DOI:** 10.1007/s11356-021-16973-x

**Published:** 2021-10-21

**Authors:** Yapeng Zhao, Xiaozhe Yin, Yan Fu, Tianxiang Yue

**Affiliations:** 1 State Key Laboratory of Resources and Environmental Information System, Institute of Geographical Sciences and Natural Resources Research, Chinese Academy of Sciences, Beijing, 100101 China; 2 College of Resources and Environment, University of Chinese Academy of Sciences, Beijing, 100049 China; 3 Department of Preventive Medicine, Keck School of Medicine, University of Southern California, Los Angeles, CA 90032 USA

**Keywords:** Plant species diversity (PSD),^.^ Remote sensing,^.^ Ensemble learning,^.^ High-accuracy surface modeling (HASM)

## Abstract

Plant species diversity (PSD) has always been an essential component of biodiversity and plays an important role in ecosystem functions and services. However, it is still a huge challenge to simulate the spatial distribution of PSD due to the difficulties of data acquisition and unsatisfactory performance of predicting algorithms over large areas. A surge in the number of remote sensing imagery, along with the great success of machine learning, opens new opportunities for the mapping of PSD. Therefore, different machine learning algorithms combined with high-accuracy surface modeling (HASM) were firstly proposed to predict the PSD in the Xinghai, northeastern Qinghai-Tibetan Plateau, China. Spectral reflectance and vegetation indices, generated from Landsat 8 images, and environmental variables were taken as the potential explanatory factors of machine learning models including least absolute shrinkage and selection operator (Lasso), ridge regression (Ridge), eXtreme Gradient Boosting (XGBoost), and Random Forest (RF). The prediction generated from these machine learning methods and in situ observation data were integrated by using HASM for the high-accuracy mapping of PSD including three species diversity indices. The results showed that PSD was closely associated with vegetation indices, followed by spectral reflectance and environmental factors. XGBoost combined with HASM (HASM-XGBoost) showed the best performance with the lowest MAE and RMSE. Our results suggested that the fusion of heterogeneous data and the ensemble of heterogeneous models may revolutionize our ability to predict the PSD over large areas, especially in some places limited by sparse field samples.

## Introduction

Plant species diversity (PSD) is an essential component of biodiversity and composed of species richness and evenness (Mcintosh and Odum 1969). Richness takes into account individual species, while evenness represents the relative abundance of species. PSD has always been used as an important indicator of the abundance of biological resource in habitats, and has a huge effect on ecosystem functions (Cardinale et al. 2012), and ecosystem services (Dong et al. 2020; Fauvel et al. 2020; Liu et al. 2018b). The mapping of PSD, therefore, has drawn much attention (Aggemyr et al. 2018; Schuler et al. 2019; Wan et al. 2020). Unfortunately, global biodiversity is declining due to anthropogenic changes to the environment, such as global warming, over-grazing, and urban construction (Ceballos et al. 2015). Therefore, it is necessary and urgent to develop a novel model to estimate the current state of diversity, which is essential for the government planning and management.

With the rapid development of monitoring technologies, many regional and global biodiversity monitoring networks were established and applied in the research of biodiversity (Fazlioglu et al. 2020; Haase et al. 2018; Wang et al. 2020). A huge amount of observation data, generated from these monitoring networks, has improved our ability to recognize and monitor the change of PSD (Boucher et al. 2020, Moudry and Devillers 2020). Focusing on site-scale surveys, traditional field-based methods can provide valuable and high-quality data at plot scale (Mallinis et al. 2020). However, predicting species diversity over large areas still remains a challenge due to the difficulties of data acquisition (Mallinis et al. 2020; Rocchini et al. 2015), as well as the bias generated from the sampling process and strategy (Lohmus et al. 2018). Therefore, the prediction of plant species diversity over large area cannot be addressed by field surveys alone, and other related techniques should be considered (Fauvel et al. 2020).

A surge in the number of satellites for remote sensing imagery, along with the improvement of interpretation algorithms, has revolutionized our ability to predict the PSD over large areas (Fauvel et al. 2020, Li et al. 2018, Melin et al. 2019, Wang &Gamon 2019). Recent studies have shown that PSD is associated with spectral bands and vegetation indices, such as Normal Difference Vegetation Index (NDVI) (Graf et al. 2019; Pearson et al. 2020), Enhanced Vegetation Index (EVI) (de Moura et al. 2017; Radeloff et al. 2019), and texture information (Fundisi et al. 2020). Owing to its superiority in spatial coverage, temporal consistency, and acceptable cost, remote sensing technology, especially in the field of earth observation, has demonstrated great potential in the prediction of PSD (Cerrejon et al. 2020; Gholizadeh et al. 2019; Rocchini et al. 2019).

Furthermore, PSD is widely known to be closely associated with climatic variables (Harrison 2020), topographic factors (Qian et al. 2020), and aboveground biomass (AGB) (Ali et al. 2019; Con et al. 2013). Tremendous efforts have been made for the establishment of their relationship; however, there is no uniform conclusion. For example, it was found that temperature showed great potential to be used as an indicator to plant diversity (Hamberg et al. 2020; McFadden et al. 2019), and some unimodal curves were confirmed (Gu et al. 2020). Nevertheless, a weak relationship was also confirmed between the temperature and PSD (Ye et al. 2020). PSD showed dramatic differences in sensitivity to temperature due to different research regions and scales.

Linear models were often used to find the relationship between PSD and these factors (Peng et al. 2019; Tsiftsis et al. 2019). It is doubtful whether multiple linear regression models can meet the actual requirements due to the multicollinearity among variables, as well as the spatial and temporal heterogeneity. To overcome the problem of collinearity, regularization methods, such as least absolute shrinkage and selection operator (Lasso) and ridge regression (Ridge), were adopted for the prediction of diversity (Kwon et al. 2018; Robinson et al. 2018). However, PSD is determined by a variety of complicated interactive factors, and could not predicted well by using these linear regression models. Therefore, it poses a new challenge to understand the relative contribution of these interactive factors.

Contrary to linear models, many non-linear regression models were also developed to establish the complicated relationship between PSD and their potential factors (Guisan and Thuiller 2005; Dufour et al. 2006; Austion et al. 2007). Machine learning, especially non-liner models, have become the most successful models in the field of remote sensing (Illarionova et al. 2021; Liu et al. 2018a). More notably, ensemble learning algorithms combine multiple different models into one stronger model so that these methods can achieve higher accuracy than a single weak learner (Guo et al. 2020; Pham et al. 2021). As the typical ensemble learning algorithms, eXtreme gradient boosting (XGBoost), and random forest (RF) can establish a non-linear relationship, but also select the relative important factors. Most importantly, they are also suitable for small datasets besides large-scale datasets (Mallinis et al. 2020; Wu et al. 2020). Therefore, they have become the most representative tree-based ensemble learning models and were selected to predict the spatial distribution of plant species diversity.

The mathematical surface is uniquely defined by the intrinsic and extrinsic properties in terms of the fundamental theorem of surfaces (Somasundaram, 2005). In the modeling of eco-environmental surface, the intrinsic properties can be collected from local information, which might come from detailed ground observations. The extrinsic properties can be gathered from global information, including satellite observations and the simulation results of spatial models on large scales. Considering the extrinsic information and intrinsic information of the surface, high accuracy surface modeling (HASM) was developed for the task of eco-environmental surface modeling (Yue et al. 2007; Yue et al. 2020). HASM has shown great potential in many applications, including temperature, precipitation, forest carbon storage, and AGB (Yue et al. 2020; Zhao et al. 2018; Zhou et al. 2021). Simulation results from different predictors and ground observations, therefore, were fused by HASM to get a high-accuracy surface of PSD.

The mapping of diversity is the primary task of biodiversity assessment, and can provide scientific support for any sector involved in biodiversity conservation and decision-making. Previous studies have shown that PSD is closely associated with environmental factors and spectral bands (Madonsela et al. 2017; Vila-Vicosa et al. 2020). Therefore, the objectives of this paper were as follows: (1) explore and identify the factors that have a great impact on the spatial distribution of PSD; (2) demonstrate the feasibility of our proposed ensemble learning models for the mapping of PSD in a large area with sparse data, especially the places that are hard and costly to reach for human beings; (3) map PSD using eight different machine learning algorithms combined with HASM fused in situ observations and remote sensing images in the first time.

## Materials and methods

### Study area

**Fig. 1:**
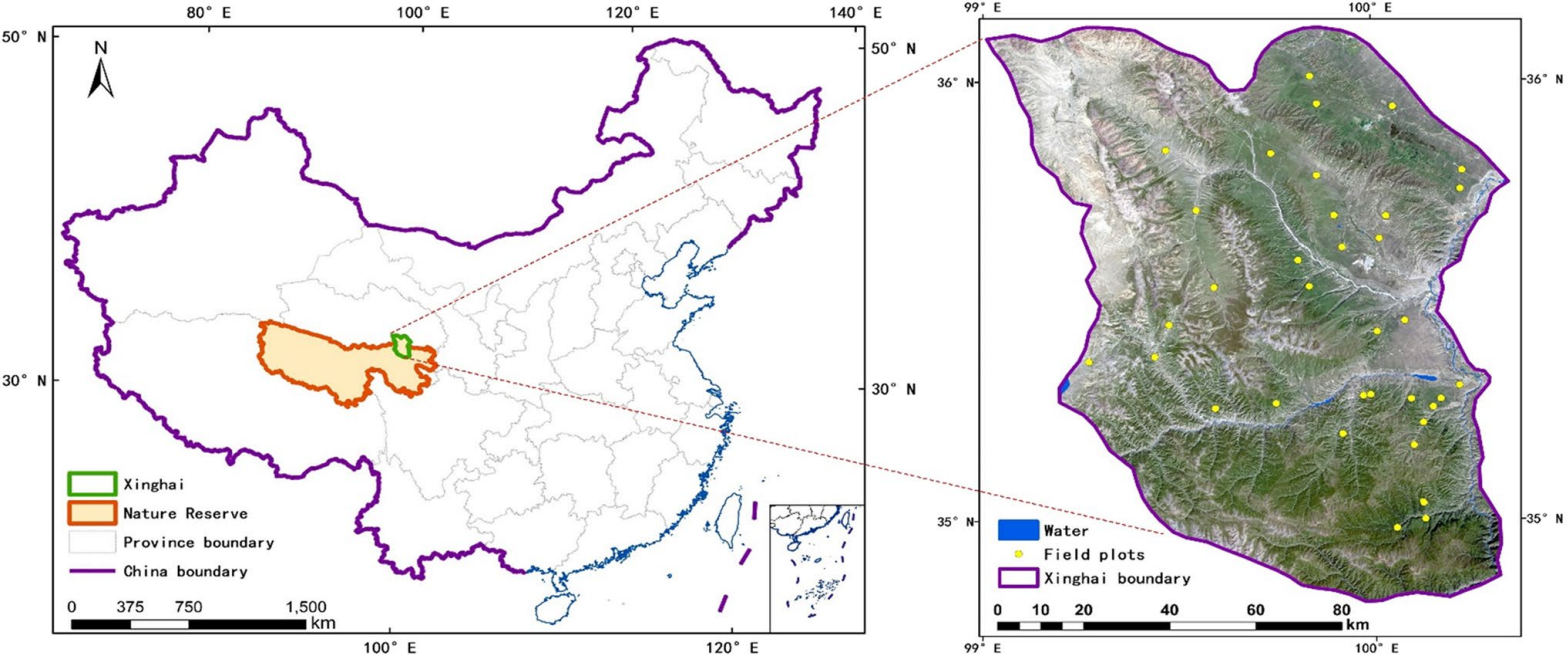
Geographical location of Xinghai

Xinghai is located in the northeast of Qinghai-Tibetan Plateau, China, and covers an area of more than 1.21 × 10^4^ km^2^ (Fig. 1), which is the core area of National Natural Reserve of Three Rivers source. The study area has a latitudinal stretch from 34°48′ N to 36°14′ N and longitudinal stretch from 99°01′ E to 100°59′ E. As a typical plateau, it is characterized by hilly and steep slopes, and the average elevation is approximately 3924 m. The climate is a typical plateau continental climate with a mean annual temperature approximately 1.8℃ and mean annual precipitation 626.2 mm. The vegetation type is dominated by grassland, as well as a small amount of woodland. Unique geographical environment and anthropogenic changes to environment make Xinghai an extremely fragile ecological area. Moreover, the ubiquitous microclimate, caused by huge altitude and terrain differences, has a great impact on the normal growths of plants at different levels, and thus leads to a huge difference in the spatial distribution of plant species diversity.

### Sampling and field surveys

According to high-resolution satellite images and gradient of topography, more than 40 plots were preselected so as to cover all vegetation types in this area. During our process of collecting data, some locations were removed because some places are hard to reach. Finally, a total of 36 sample plots, with different elevations and habitats, were selected and collected in August and September, 2019 (Fig. 1). For the sampling in grassland, 3 different and representative quadrats of 1 m^2^ were recorded within each plot. Meanwhile, plant species, coverage, average height, and habitat information within each plot were recorded during field survey. We measured AGB (including litter) of each quadrat by clipping its aboveground plant debris. All collected samples were oven-dried at 70℃ for more than 24 h and weighted with a precision of 0.01 g in the laboratory. As for the sampling in forest, all species information was collected and calculated within each plot in a similar way except the measurement of AGB.

### Satellite image and environmental data

Landsat 8 Operational Land Imager (OLI) sensor satellite images were acquired from USGS (United States Geological Survey). We selected all the atmospherically corrected surface reflectance from May to October in the year of 2018, 2019, and 2020 using the platform of Google Earth Engine. Every scene records a coastal, a blue, a green, a red, and a near-infrared band, as well as two shortwave-infrared bands at a spatial resolution of 30 m × 30 m. Considering the limitations imposed by cloud cover and the satellite’s temporal resolution (16 days), these satellite images were selected and merged into a complete image according to its maximum of NDVI.

There were many studies that confirmed that PSD was related with vegetation indices (Schmidtlein &Fassnacht 2017, Torresani et al. 2019; Vila-Vicosa et al. 2020). Based on the former merged Landsat 8 images, six common vegetation indices were chosen and calculated considering multi-variable collinearity, and then incorporated into the prediction of PSD (Table 1).

**Table 1:** Vegetation indices included in this study

Vegetation index	Formula	Reference
Normalized Difference Vegetation Index (NDVI)	$\mathrm{NDVI}=\frac{{\rho }_{\mathrm{NIR}}-{\rho }_{\mathrm{red}}}{{\rho }_{\mathrm{NIR}}+{\rho }_{\mathrm{red}}}$	(Huete et al. 2002)
Enhanced Vegetation Index (EVI)	$\mathrm{EVI}=\mathrm{G}\frac{{\rho }_{\mathrm{NIR}}-{\rho }_{\mathrm{red}}}{{\rho }_{\mathrm{NIR}}+{\mathrm{C}}_{1}\times {\rho }_{\mathrm{red}}-{\mathrm{C}}_{2}\times {\rho }_{\mathrm{blue}}+L}$	(Huete et al. 2002)
Normalized Difference Water Index (NDWI)	$\mathrm{NDWI}=\frac{{\rho }_{\mathrm{NIR}}-{\rho }_{\mathrm{SWIR}1}}{{\rho }_{\mathrm{NIR}}+{\rho }_{\mathrm{SWIR}1}}$	(Hardisky et al. 1983)
Carotenoid Reflectance Index (CRI)	$\mathrm{CRI}=1/{\rho }_{\mathrm{blue}}-1/{\rho }_{\mathrm{green}}$	(Gitelson et al. 2002)
Simple Ratio Index (SRI)	$\mathrm{SRI}={\rho }_{\mathrm{NIR}}/{\rho }_{\mathrm{red}}$	(Birth and Mcvey 1968)
Difference Vegetation Index (DVI)	$\mathrm{DVI}={\rho }_{\mathrm{NIR}}-{\rho }_{\mathrm{red}}$	(Tucker 1979)

In addition to the Landsat data, AsterDem data were provided by Geospatial Data Cloud site, Computer Network Information Center, Chinese Academy of Sciences, which were used for the extraction of elevation with a spatial resolution of 30 m × 30 m. Thus, slope, aspect, and curvature were obtained from it using the geomorphometry toolbox.

Based on an improved downscaling method (Zhao et al. 2018), precipitation (mean annual precipitation) was produced according to observation data from the national meteorological stations and its environmental factors. And temperature (mean annual temperature) was collected and downscaled from the Resource and Environment Science and Data Center, Institute of Geographic Sciences and Natural Resources Research, CAS, and National Meteorological Science Data Center. AGB was calculated from the field surveyed observations and its environmental variables using HASM (Zhou et al. 2021).

### Methodology

**Fig. 2:**
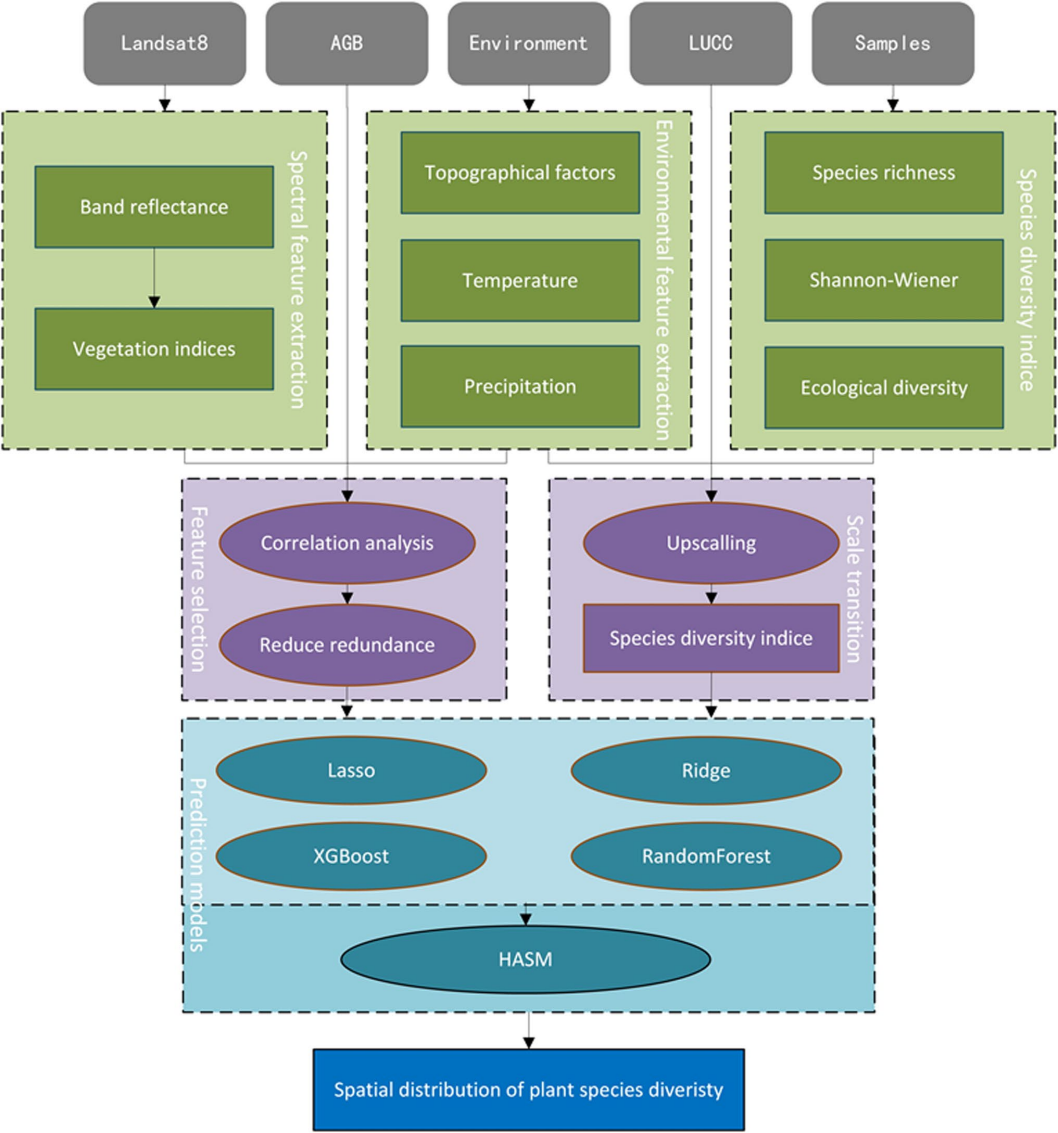
Flowchart of the mapping of PSD using our proposed ensemble learning model

Ensemble models, machine learning models combined with HASM, were proposed for the task of mapping of PSD from remote sensing images and environmental factors (Fig. 2). First, feature extraction and diversity index calculation were conducted based on the Landsat images and sampling data. Then, we continued the process of feature selection and scale transition according to the extracted features.

Furthermore, potential explanatory features, including spectral reflectance, vegetation indices, and environmental variables, were taken as the input of linear regression and ensemble learning models. Finally, HASM was used as the optimum control model to reduce the gap between the predictions of former models by using the survey data. More details of our proposed new ensemble models are illustrated in the following section.

Three species diversity indices were used to describe the statistical characteristics of PSD in this study. Species richness (SR), Shannon index (SH), and ecological diversity (ED) (Yue et al. 2007) were calculated from the field surveys. Species richness represents the total number of plant species that occurred within each plot and is defined as (Magurran et al. 1988):

1 $$S=\frac{n}{A}$$

where $\mathrm{n}$ is the number of species and $\mathrm{A}$ is the area of the plot.

Shannon index is usually expressed by H’, and it is defined as:

2 $$\mathrm{H}'=-{\sum}_{i=1}^n{p}_i\ln {p}_i$$

where $\mathrm{n}$ is the number of species and ${\mathrm{p}}_{\mathrm{i}}$ is the proportional cover of the *i*_th_ species.

ED is a representative index of plant species richness and evenness, and its index is defined as (Yue et al. 2007):

3 $$\mathrm{ED}=\frac{{\mathrm{ln}(\sum_{i=1}^{n}{({p}_{i})}^\frac{1}{2})}^{2}}{\mathrm{ln}\varepsilon }$$

where $\upvarepsilon ={(e+A)}^{-1}$ , $e$ is a constant of 2.71828, and $\mathrm{A}$ is the area of the quadrat.

To predict the PSD, two traditional linear regression methods (Lasso and Ridge) and two representative ensemble learning algorithms (XGBoost and RF) were used to build the function between diversity and its factors. HASM was then employed to optimize the residual between the predicted function and their observed value. Thus, a total of eight methods were used to predict the PSD in our study including linear regression models, ensemble learning models, and HASM-based models (HASM-Lasso, HASM-Ridge, HASM-XGBoost, and HASM-RandomForest).

Multiple linear regression (MLR) models have been widely applied in predicting plant species diversity using environmental and spectral factors. One of the assumptions is that there is no linear relationship among explanatory variables while using MLR models (Mallinis et al. 2020). Unfortunately, multicollinearity is a prevalent problem in the linear regression models solved by ordinary least square (OLS) methods. It is necessary to consider the dimension reduction to avoid the feature redundancy. To avoid the problems of data redundance or multicollinearity, regularization was used before modeling, so that the variables with high correlation with other input factors will be discarded before the linear regression model solved by ordinary least square (OLS) methods. Regularization was adopted in the prediction process to avoid the overfitting caused by insufficient sampling data and redundant features. For the supposed dataset $D\{{(x}_{1},{\mathrm{y}}_{1}),{(x}_{2},{\mathrm{y}}_{2}),\dots ,{(x}_{m},{\mathrm{y}}_{m})\}$ , the optimization objective of MLR was usually defined as:

4 $$\underset{w}{\mathrm{min}}\sum_{i}^{m}{({y}_{i}-{w}^{T}{x}_{i})}^{2}$$

where $x\in {\mathbb{R}}^{d}$,$y\in {\mathbb{R}}$, $w=({w}_{1};{w}_{2};\dots ;{w}_{d})$ . The problem of overfitting will be inevitable if there are sufficient sample features without corresponding samples, which will lead to the saturation of prediction accuracy, even a sharp decline. Therefore, $\mathrm{L}2-\mathrm{norm}$ was introduced in Ridge regression to reduce overfitting, and the optimization objective was shown as (Tikhonov et al. 1977):

5 $$\underset{w}{\mathrm{min}}\sum_{i}^{m}{({y}_{i}-{w}^{T}{x}_{i})}^{2}+\uplambda {\Vert w\Vert }_{2}^{2}$$

where $\uplambda >0$. $\mathrm{L}1-\mathrm{norm}$ was adopted in Lasso, and its optimization objective was redefined as (Tibshirani, 1996):

6 $$\underset{w}{\mathrm{min}}\sum_{i}^{m}{({y}_{i}-{w}^{T}{x}_{i})}^{2}+\uplambda {\Vert w\Vert }_{1}$$

$\mathrm{L}1-\mathrm{norm}$ can not only reduce overfitting, but also obtain sparse features. Therefore, some redundant features would be discarded if they had a strong correlation with other features.

Although various linear regression methods had been identified to be effective, it is still a challenge for the complicated non-linear relationship due to the spatial and temporal heterogeneity. XGBoost was an improved version of gradient boosting algorithm and has produced state-of-the-art results in ecological applications (Li et al. 2021; Luo et al. 2021). Considering random subsets of features and sample data, RF showed a better performance than the other bagging methods in the generalization error. RF and XGBoost have become the most representative models of bagging and boosting, respectively.

RF and XGBoost were implemented using the ensemble and xgboost package in Python. Their parameters were optimized by using the package of GridSearchCV in Python instead of tedious and time-consuming manual adjustments. Re-weighting and bootstrap sampling were the major differences between XGBoost and Random Forest in respect of sampling. All environmental and remote sensing factors were used as potential explanatory variables. XGBoost and RF were capable of selecting the relatively important factors in the prediction process.

According to the fundamental theorem for eco-environmental surface modeling (FTEEM), an eco-environmental surface is uniquely defined by the intrinsic and extrinsic information (Yue et al. 2020). In the prediction of PSD, the intrinsic information comes from the diversity values of sampling data, and the extrinsic from the regression results of the machine learning algorithms. In the process of prediction, HASM is used to integrate the above information and can be seen as a data fusion method instead of an interpolator. The detailed equations of HASM have been published in previous articles ( Yue et al. 2007); therefore, its main computational process is illustrated here. The computational formula of HASM, solving the following equality-constrained least squares problem, can be expressed as (Yue 2011, Zhao et al. 2018):

7 $$\left\{\begin{array}{c}{min\Vert \left[\begin{array}{c}A\\ B\\ C\end{array}\right]\bullet {x}^{n+1}-\left[\begin{array}{c}d\\ q\\ h\end{array}\right]\Vert }_{2}\\ S\bullet {x}^{n+1}=k\end{array}\right.$$

where $A$, $B$, and $C$ are the coefficient matrices of the first equation, the second equation, and the third equation in Gauss equations, respectively (Toponogov 2006). $d$,$q,$ and $p$ are the right-hand vectors of the former three equations, respectively. $n$ is the number of iterations. $S$ is the coefficient matrix of observation data, and $k$ represents the values of sampling matrix.

**Fig. 3:**
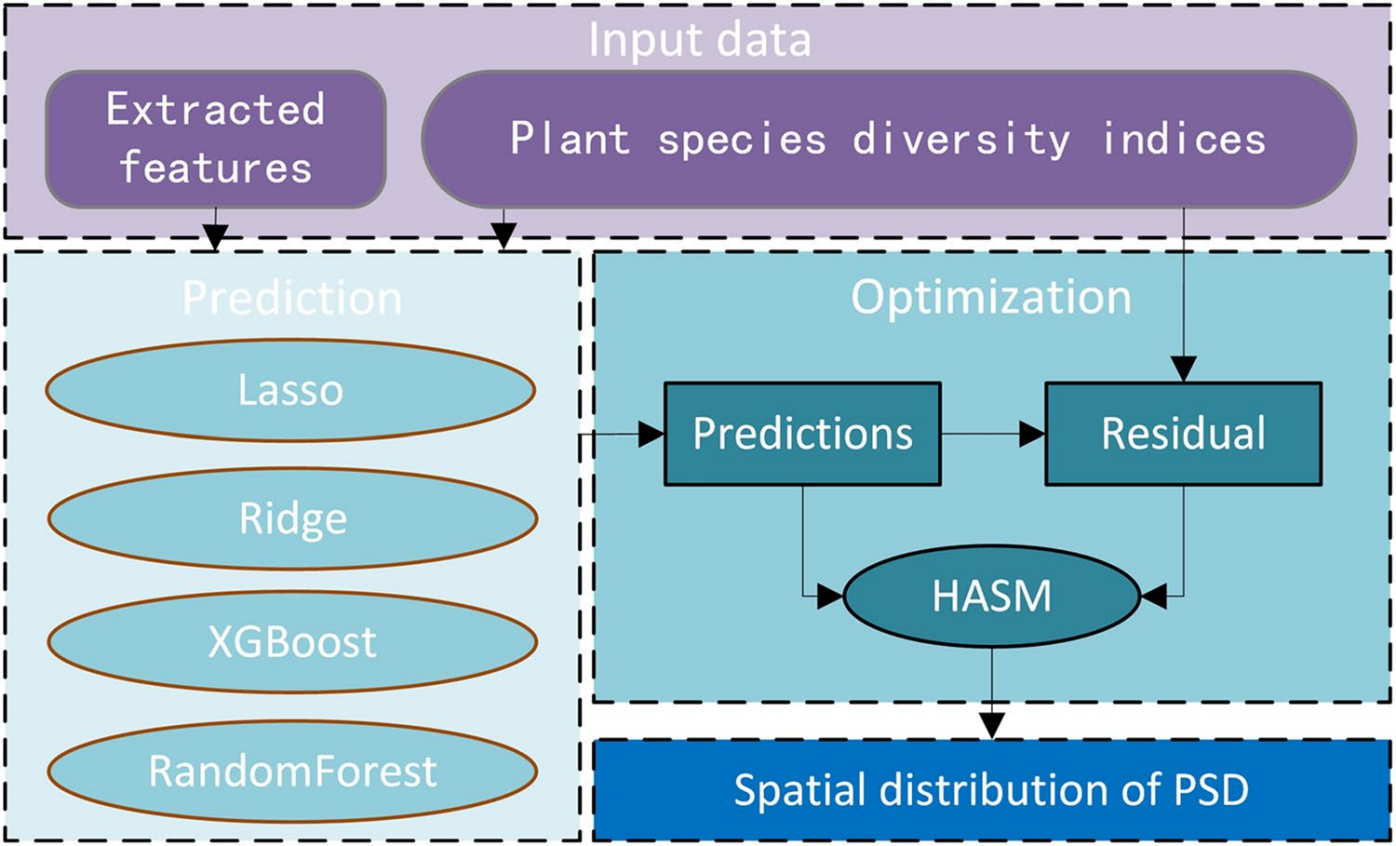
The prediction and optimization for the simulation of PSD

After the prediction of linear regression and ensemble learning models, the prediction and its residual with the observed value of diversity indices were taken as the input of HASM. Finally, spatial distribution of PSD was generated according to the prediction and recalculated residual (Fig. 3).

### Accuracy assessment

To evaluate the performance of different models, 90% of the sample points were selected randomly as training data, and the remaining 10% samples were taken as validation data. Their mean absolute error (MAE) and root mean square error (RMSE) were calculated after the above process were repeated 10 times considering the insufficient data and outliers in our sampling. In addition, MAE and RMSE is usually defined as:

8 $$\mathrm{MAE}=\frac{1}{m}\sum_{i=1}^{m}\left|h\left({x}_{i}\right)-{y}_{i}\right|$$

9 $$\mathrm{RMSE}=\sqrt{\frac{1}{m}\sum_{i=1}^{m}{(h\left({x}_{i}\right)-{y}_{i})}^{2}}$$

where $m$ is the number of the validation dataset, $h({x}_{i})$ and ${y}_{i}$ are the predicted and observed values of the *i*th sampling data, respectively.

## Results

### Relationships between plant species diversity and explanatory variables

The statistics of three PSD indices of the sampling plots are shown in Table 2. The values of SR ranged from 10 to 31, and its absolute value of skewness (skew) was much lower than the absolute value of other two indices. SH had the worst of skewness and kurtosis (kurt). Compared with SR and SH, ED had the best performance in terms of kurtosis (kurt = 2.51), and a relatively lower skewness than SH. Moreover, ED showed a significant and positive correlation with SR and SH, and the correlation coefficients were 0.88 and 0.98, respectively, which were higher than the correlation between SR and SH (0.79). This also verified the hypothesis that the ED index could represent the information of species richness, as well as species evenness.

**Table 2:** Statistical analysis of different plant species diversity

Indices	Min	Max	Mean	Std	Skew	Kurt
SR	10	31	19.31	4.91	0.27	2.59
SH	1.00	2.79	2.12	0.43	− 0.70	3.03
ED	1.11	2.31	1.82	0.30	− 0.55	2.51

PSD showed a significant and positive correlation with NDVI, EVI, NDWI, SRI, DVI, and NIR (*r* > 0.45), and a negative correlation with spectral bands, except NIR (Table 3). Elevation, aspect, slope, plain curvature (PlainCure), profile curvature (ProfileCure), AGB, temperature, precipitation, and CRI showed weaker correlation with plant diversity indices. SR showed stronger correlation with the Landsat images and environmental factors compared with SH and ED. NDVI and SRI have the highest correlation with SR (*r* = 0.68; *p* < 0.001).

**Table 3:** Pearson correlation coefficient between species diversity indices and explanatory variables

Variables	SR	SH	ED
NDVI	0.68***	0.55***	0.62***
SRI	0.68***	0.53***	0.61***
NDWI	0.66***	0.53***	0.6***
EVI	0.66***	0.47**	0.54***
DVI	0.64***	0.55***	0.6***
SWIR2	− 0.62***	− 0.47**	− 0.54***
Blue	− 0.62***	− 0.46**	− 0.55***
Red	− 0.63***	− 0.45**	− 0.53***
Coastal	− 0.59***	− 0.44**	− 0.52***
Green	− 0.57***	− 0.39*	− 0.48**
SWIR1	− 0.53***	− 0.37*	− 0.44**
NIR	0.47**	0.45**	0.48**
Temperature	0.43**	0.23	0.31
AGB	0.37*	0.26	0.32
Elevation	− 0.31	− 0.17	− 0.21
Aspect	− 0.27	− 0.19	− 0.23
Slope	0.25	0.18	0.21
Precipitation	0.19	0.13	0.13
ProfileCure	0.16	0.21	0.18
CRI	− 0.15	0.04	− 0.01
PlainCure	− 0.13	− 0.11	− 0.11

^***^p < 0.001, **p < 0.01, *p < 0.05

### Comparative analysis of the mapping generated from different methods

According to the process of validation described in the former section, validation datasets were used to test the performances of different models. MAE and RMSE of each model were calculated after this process was repeated 10 times, and shown in Table 4. It was noted that some high observed diversity indices were underestimated, whereas some low observed values were overestimated among all single regression models and ensemble learning models. After the fusing of HASM, HASM-based methods (HASM-Lasso, HASM-Ridge, HASM-XGBoost, and HASM-RandomForest) showed better performance than the regression models (Lasso and Ridge) or ensemble learning models (XGBoost and RF) with lower MAE and RMSE values; thus, the predicted values were closer to the observed values of plant diversity indices.

**Table 4:** The comparisons of the prediction accuracy by different methods

Methods	MAE			RMSE		
	SR	SH	ED	SR	SH	ED
Lasso	2.96	0.30	0.19	3.65	0.38	0.25
Ridge	2.85	0.30	0.19	3.46	0.37	0.24
XGBoost	0.93	0.13	0.12	1.26	0.19	0.16
RandomForest	1.50	0.18	0.11	1.94	0.25	0.14
HASM-Lasso	1.98	0.28	0.08	2.73	0.35	0.12
HASM-Ridge	2.49	0.18	0.17	3.12	0.26	0.21
HASM-XGBoot	**0.89**	**0.07**	**0.06**	**1.19**	**0.12**	**0.10**
HASM-RandomForest	1.12	0.12	**0.06**	1.57	0.20	**0.10**

Lasso had the worst performance with the highest values of MAE (SR, 2.96; SH, 0.30; ED, 0.19) and RMSE (SR, 3.65; SH, 0.38; ED, 0.25) among all methods, followed by the Ridge methods. For the ensemble learning methods, XGBoost showed a better performance with lower MAE and RMSE in terms of SR and SH, whereas RF had a slightly higher accuracy in the indices of ED. It is noted that ensemble learning models showed better accuracy than regression methods.

Compared with regression or ensemble learning methods, HASM-based methods had much better accuracy with lower MAE and RMSE. HASM-XGBoost showed the best performance with the lowest MAE (SR, 0.89; SH, 0.07; ED: 0.06) and RMSE (SR, 1.19; SH, 0.13; ED, 0.10), followed by HASM-RandomForest. And ensemble learning models combined with HASM also had a better performance than the regression methods combined with HASM, which was consistent with the single regression or ensemble learning models.

### Mapping of plant species diversity

**Fig. 4:**
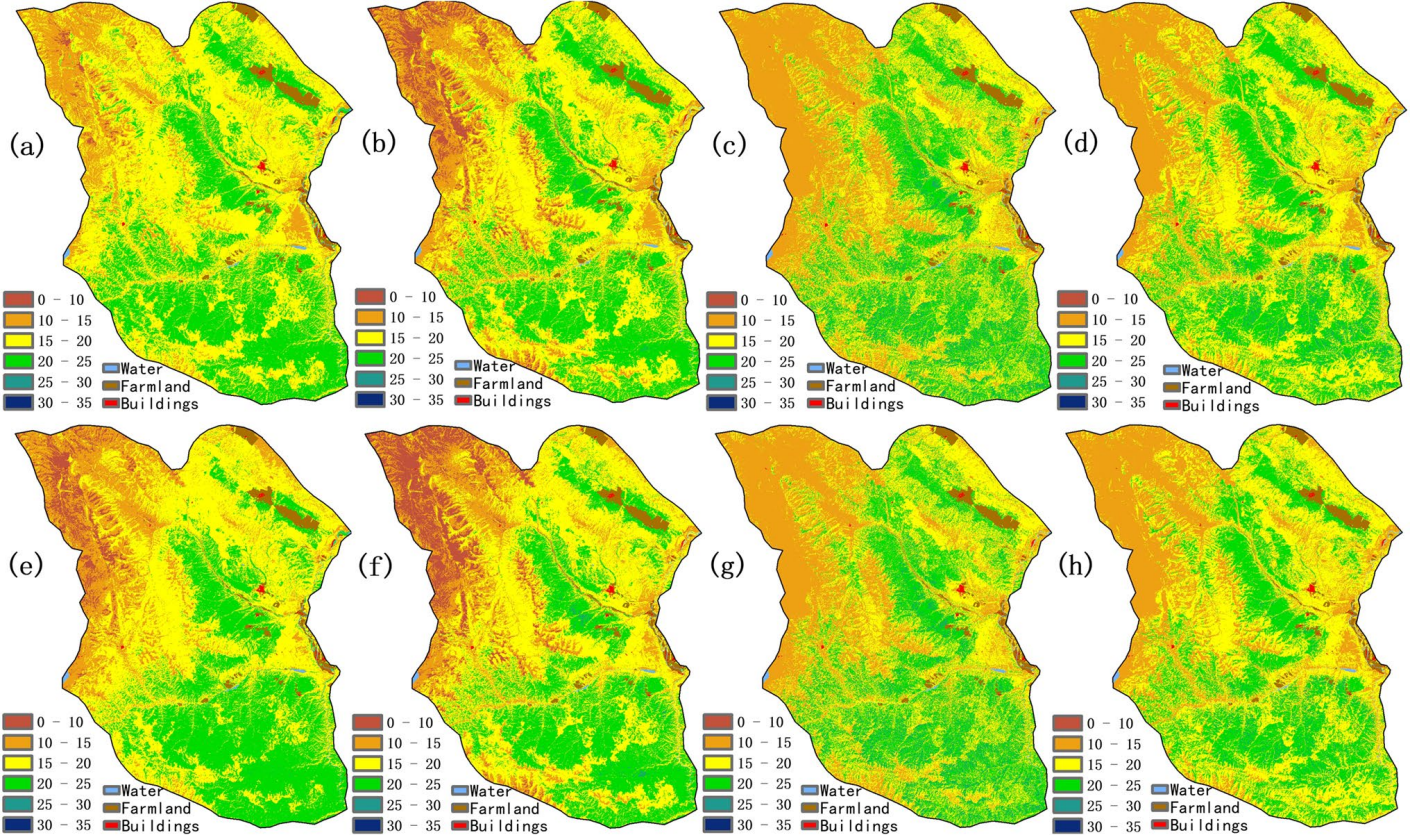
The prediction maps of species richness: (**a**) Lasso, (**b**) Ridge, (**c**) XGBoost, (**d**) Random Forest, (**e**) HASM-Lasso, (**f**) HASM-Ridge, (**g**) HASM-XGBoost, (**h**) HASM-RandomForest

**Fig. 5:**
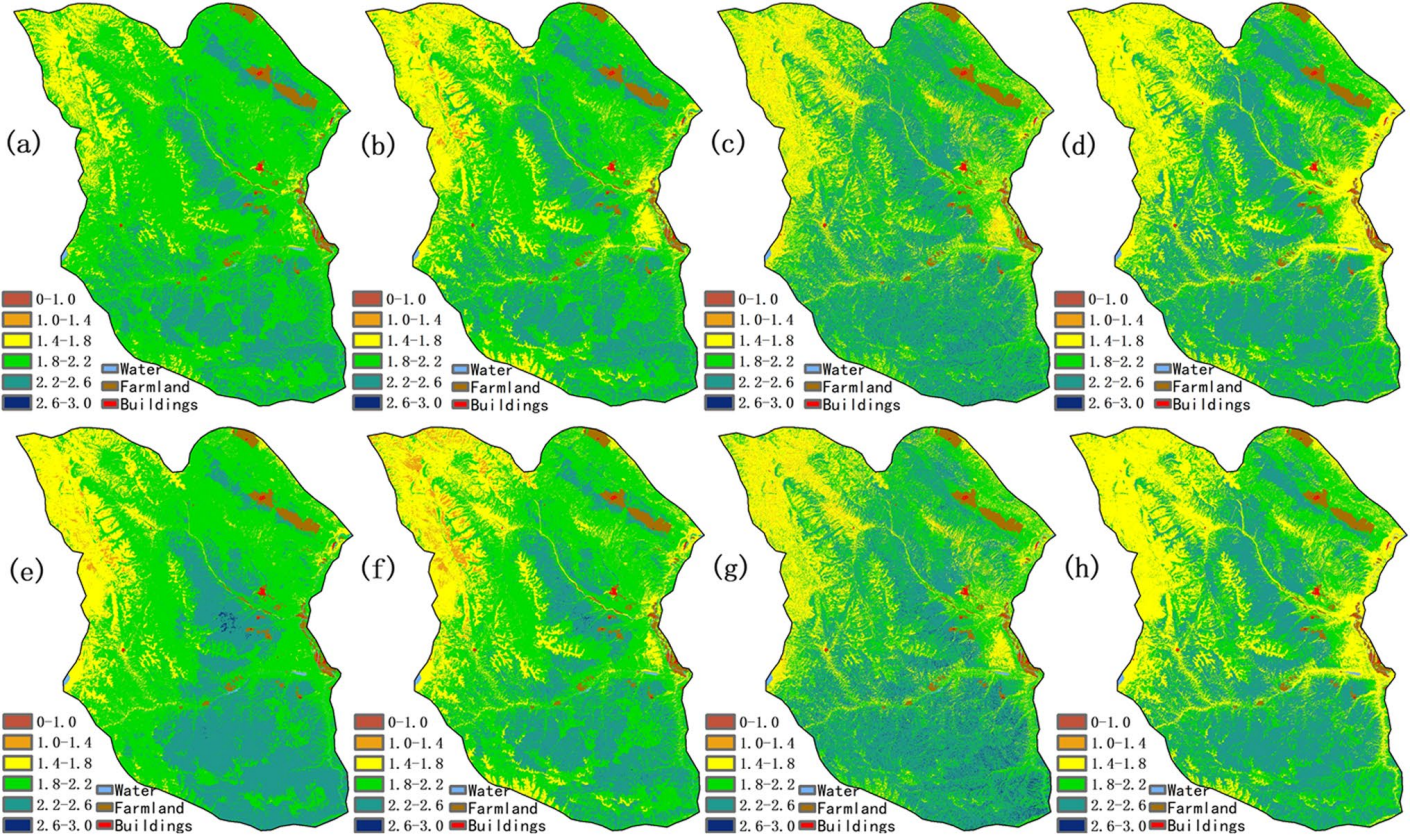
The prediction maps of Shannon index: (**a**) Lasso, (**b**) Ridge, (**c**) XGBoost, (**d**) Random Forest, (**e**) HASM-Lasso, (**f**) HASM-Ridge, (**g**) HASM-XGBoost, (**h**) HASM-RandomForest

**Fig. 6:**
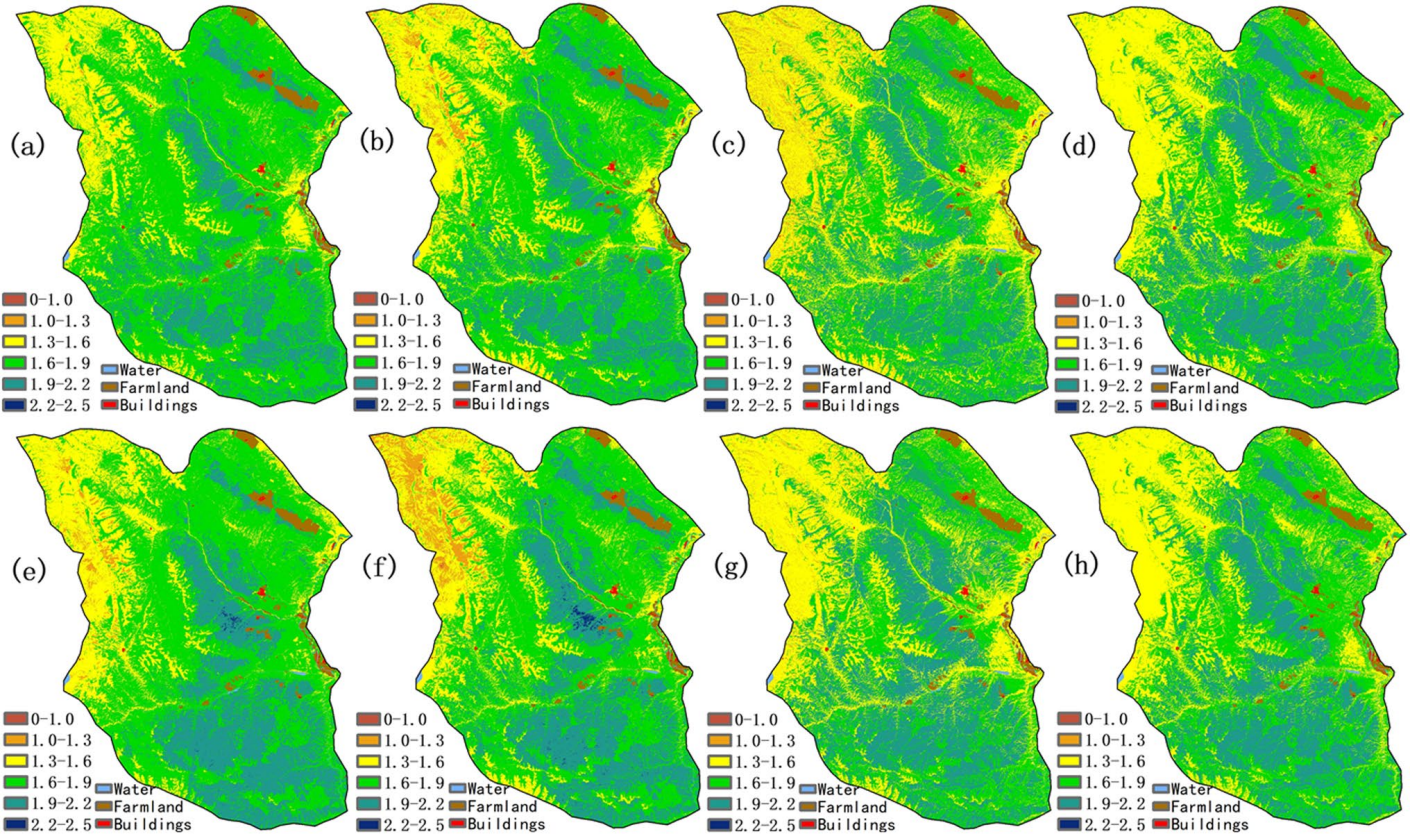
The prediction maps of ecological diversity: (**a**) Lasso, (**b**) Ridge, (**c**) XGBoost, (**d**) Random Forest, (**e**) HASM-Lasso, (**f**) HASM-Ridge, (**g**) HASM-XGBoost, (**h**) HASM-RandomForest

The predictions of SR, SH, and ED, generated from eight different models, are shown in Figs. 4, 5, and 6, respectively. Ensemble learning models showed a similar spatial distribution pattern, but were slightly different with the regression models because there were not sufficient sampling points, especially in the northwest region. Species diversity had a significant correlation with the spectral bands and its vegetation indices and were affected by environmental factors.

For the prediction of SR, high values of SR (> 20) were mainly distributed in the east and southeast region with high NDVI (> 0.14), SRI (> 1.32), AGB (> 72 g/m^2^), temperature (> − 6.4℃), and precipitation (> 451 mm) at an elevation below 4700 m (Fig. 4). Some low values of species richness below 10, generated mainly from the regression models, were located in the northwest area with high elevation and low NDVI (< 0.32). After fusing the HASM, ensemble learning models showed the similar distribution in spite of some details, while it is obvious that the proportion of values less than 10 was much higher than the simple regression methods in the northwest area.

For SH, regression models and ensemble learning models showed a similar spatial distribution pattern. High values of SH (> 2.2) were distributed in the east and southeast region with higher NDVI (> 0.33), SRI (> 2.0), AGB (> 74 g/m^2^), precipitation (> 455 mm), temperature (> − 7.9℃), and lower elevation (< 4700 m) (Fig. 5). Low values of SH were distributed in the northwest area with low NDVI and SRI, and high elevation, which is similar with the distribution pattern of SR. More obvious distinguishment emerged in the results between Lasso and HASM-Lasso models in the northwest, and the other HASM-based models showed a slight difference compared with single models.

In terms of ED, high values of ecological diversity (> 2.0) were distributed in the east and southeast region with higher NDVI (> 0.43), SRI (> 2.5), AGB (> 102 g/m^2^), precipitation (> 523 mm), temperature (> − 6℃), and lower elevation (< 4458 m) (Fig. 6). The values generated from Ridge, Lasso, and Ridge models combined with HASM were lower than other models in the northwest area. Some high values of ED (> 2.2) appeared in the prediction of regression models combined with HASM.

## Discussion

The significant relationship of PSD with spectral bands and vegetation indices suggested that satellite images could be used as important indicators of species diversity. Landsat 8 spectral bands except the NIR bands showed a negative relationship with PSD (Madonsela et al. 2017), which may be related to the strong reflection of NIR and the absorption of visible light in the process of photosynthetically active radiation (RAR). Unlike the spectral reflectance, vegetation indices focused on the variability from vegetation characteristics by suppressing the spectral reflectance from non-vegetation features (Huete et al. 2002; Vina et al. 2011). Furthermore, our study confirmed their significant positive relationship with species diversity, including NDVI (Tibshirani 1996; Vina et al. 2011), EVI (Cabacinha and de Castro 2009), NDWI (Vila-Vicosa et al. 2020), and DVI (Hashemi et al. 2013). It is not surprising that a combination of vegetation indices and spectral reflectance improves the ability to predict the PSD. PSD showed a significant relationship with spectral bands and vegetation indices in this study area. However, it will change dramatically when dealing with another different area; for example, NDVI might not be a good indicator, while ANPP or NPP has a negative correlation with diversity in wetter ecosystems.

Moreover, species diversity was observed to be sensitive to environmental variables such as temperature, precipitation, topography, and aboveground biomass (Vila-Vicosa et al. 2020). Contrary to the weak relationship (Waide et al. 1999), elevation showed a significant negative relationship with species diversity in the study area. Other studies also found some different relationships including U-shaped and hump-shaped relationship (Bassler et al. 2016; Mapfumo et al. 2016; Nagendra et al. 2013). One possible explanation for these unclear relationships may be their unique complicated interaction in different area, e.g., elevation, that had a different significant impact on local temperature, precipitation, potential evapotranspiration, pressure, and the length of growing season. As a consequence, species diversity showed a difference in sensitivity to elevation and its interactive climatic factors in different places.

We compared the performance of different predictors including regression models and ensemble learning models. Compared with regression models, XGBoost and RF models showed a higher accuracy with lower MAE and RMSE. Random Forest had proven its strong explanatory ability to obtain accurate predictions of species diversity with limited field samples (Cabezas et al. 2016; Laurin et al. 2014). One possible explanation may be their ability to handle non-linear relationship for complicated interactive environmental variables and strong robustness for small-scale dataset (Mallinis et al. 2020; Wu et al. 2020). XGBoost, a scalable tree boosting system, were employed to achieve many state-of-the-art challenges in previous studies (Chen and Guestrin 2016). It is therefore not surprising that XGBoost showed slightly better results than the regression models.

Machine learning algorithms such as RF and XGBoost have been demonstrated to be an effective tool for modeling species diversity. Nevertheless, it was inevitable that low values of species diversity were overestimated and high values were underestimated among machine learning models. HASM has been developed for the task of eco-environmental surface modeling and achieved satisfactory results in many aspects, such as elevation, climate, XCO2, and aboveground biomass (Yue et al. 2019; Zhao et al. 2019; Zhou et al. 2021). Therefore, a novel ensemble learning model combined with HASM was proposed for the mapping of PSD. After the fusing of HASM, ensemble models had a better performance than the former models, especially the HASM-XGBoost model. Possible explanations for such a better performance of HASM-XGBoost may be (i) HASM-XGBoost had a stronger ability in dealing with non-linear relationship and was also available for small dataset besides large-scale dataset; (ii) the deficiency of XGBoost, low values were overestimated and high values were underestimated, was made up by the novel ensemble model. A perfect combination of statistics and geometric analysis significantly has improved the performance of the HASM-XGBoost in the prediction of PSD.

These diversity maps can provide scientific data and guidance for the local authorities involved in biodiversity assessment and decision-making. More importantly, the proposed ensemble model can enhance our ability to predict the spatial distribution of PSD in a large area, especially in some places limited by sparse field samples. Meanwhile, the ensemble models may not have been truly explored due to the limited filed data. Therefore, more explanatory variables and training data will be used to improve the generalization capability of ensemble model in our future work.

## Conclusion

Remotely sensed variables and environmental factors were fused to predict the distribution of PSD by using machine learning algorithms combined with HASM. The study demonstrated that vegetation indices had significant positive relationship with species diversity, and a negative relationship was observed between spectral reflectance and species diversity. Although their opposite relationship, combining vegetation indices with spectral bands enhanced the explanatory power of remotely sensed images (Madonsela et al. 2017). Unlike their clear positive or negative correlation, environmental variables showed a complicated relationship with species diversity, and mainly due to their interaction between each other in different area. In conclusion, PSD is closely associated with vegetation indices, followed by spectral bands and environmental factors.

It is observed that all models were effective and could produce similar spatial distribution of PSD. However, ensemble learning models showed a better performance than regression models benefiting from its superiority in dealing with non-linear relationship and small-scale dataset, especially XGBoost. Moreover, ensemble learning models combined with HASM had higher accuracy in the prediction of species diversity, which was consistent with many applications using HASM. Among all ensemble models, XGBoost combined with HASM (HASM-XGBoost) was the best choice for the mapping of PSD.

Combining Landsat 8 satellite images and environmental variables, ensemble models combined with HASM showed a strong explanatory power in predicting the spatial distribution of PSD. The study suggested that the fusion of heterogeneous data and the ensemble of heterogeneous models will revolutionize our ability to predict the PSD in a large area with limited field samples, especially in some places that are hard and costly to reach for human beings.

## Data Availability

Not applicable.
